# Subjective Memory: A Clinical Marker for Cognitive Decline and Depression in Older Puerto Ricans With Chronic Pain

**DOI:** 10.1093/geroni/igaf122.1501

**Published:** 2025-12-31

**Authors:** Tyler Bell, Caitlin Northcutt, Sadaf Milani, Nathalie Gider, Michael Crowe

**Affiliations:** University of California, San Diego, San Diego, California, United States; University of Kentucky, Lexington, Kentucky, United States; University of Texas Medical Branch, Galveston, Texas, United States; University of California, San Diego, San Diego, California, United States; University of Alabama at Birmingham, Birmingham, Alabama, United States

## Abstract

Chronic pain has been linked to subjective memory problems in older Puerto Rican adults, yet its relationship to objective cognitive decline and depressive symptoms remains unclear. This study examined these associations using data from Waves 1 (2002/2003), 2 (2006/2007), 3 (2020/2021), and 4 (2022/2024) of the Puerto Rican Elderly: Health Conditions Study (PREHCO), a longitudinal, population-based study of Puerto Rican adults aged 60 and older. The sample included individuals who were cognitively unimpaired at baseline with at least one follow-up (n = 2,630, Mage=71.33, SD = 8.25; 59.5% female). Chronic pain was defined as bodily pain reported at both Waves 1 and 2. Subjective memory was assessed using the Health and Retirement Study item, “How would you rate your memory at the present time?” (1 = excellent to 5 = poor). Cognitive function and depressive symptoms were measured using the Mini-Mental Caban and the 15-item Geriatric Depression Scale, respectively. Structural equation modeling examined how chronic pain at Wave 2 influenced subjective memory concerns at Wave 3 and cognitive decline at Wave 4, accounting for prior cognitive function. Chronic pain was associated with worse subjective memory problems (β=.31, p<.001) and greater depressive symptoms (β=.31, p<.001) at Wave 3. By Wave 4, chronic pain predicted increases in depressive symptoms (β=-.08, p=.038). Bootstrap methods confirmed that subjective memory problems mediated the relationship between chronic pain and cognitive decline (β=-.27, p=.035) and depressive symptoms (β=.23, p=.008). These findings highlight subjective memory as a potential intermediatory step between declines in cognitive and emotional health in aging Puerto Rican adults.

